# Supplementary material for: Phase Transitions of Cu and Fe at Multiscales in an Additively Manufactured Cu–Fe Alloy under High-Pressure
Source: Nanomaterials (Basel). 2022 Apr 29;12(9):1514. doi: 10.3390/nano12091514 (PMC9104048; doi:10.3390/nano12091514)
Supplement: Supplementary file 1 [file nanomaterials-12-01514-s001.zip › nanomaterials-1669237-supplementary/Supplementary Files Description.pdf]

Videos S1, S3, S5, S7. Series of Laue diffraction patterns from crystals 1,2,3,4 respectively exhibiting gradual pressure induced deformation. Red arrows denote predicted positions of reflections with d-values above 0.5Å for crystals 1,3,4 and 0.6Å for crystal 2. The intensity scaling of the patterns is kept the same along each movie as defined in Fit2d program [1].

Videos S2, S4, S6, S8. Series of maps of Laue reflections from crystals 1,2,3,4 respectively exhibiting gradual pressure induced deformation. Red rectangles denote positions where corresponding diffraction patterns in Videos S1, S3, S5, S7 were collected. The intensity scaling of the maps is kept the same along each movie as defined in Fit2d program [1]. Blue rectangles denote area presented in Figure 7.

References

1. Hammersley, A.P.; Svensson, S.O.; Hanfland, M.; Fitch, A.N.; Hausermann, D. Two-dimensional detector software: From real detector to idealized image or two-theta scan. J. High Press. Res. 1996, 14, 235–248.
